# Supplementary material for: Identifying the factors governing internal state switches during nonstationary sensory decision-making
Source: Nat Commun. 2025 Nov 27;16:11684. doi: 10.1038/s41467-025-66738-0 (PMC12749821; doi:10.1038/s41467-025-66738-0)
Supplement: Supplementary file 1 — Supplementary Information [file 41467_2025_66738_MOESM1_ESM.pdf]

Supplementary Information

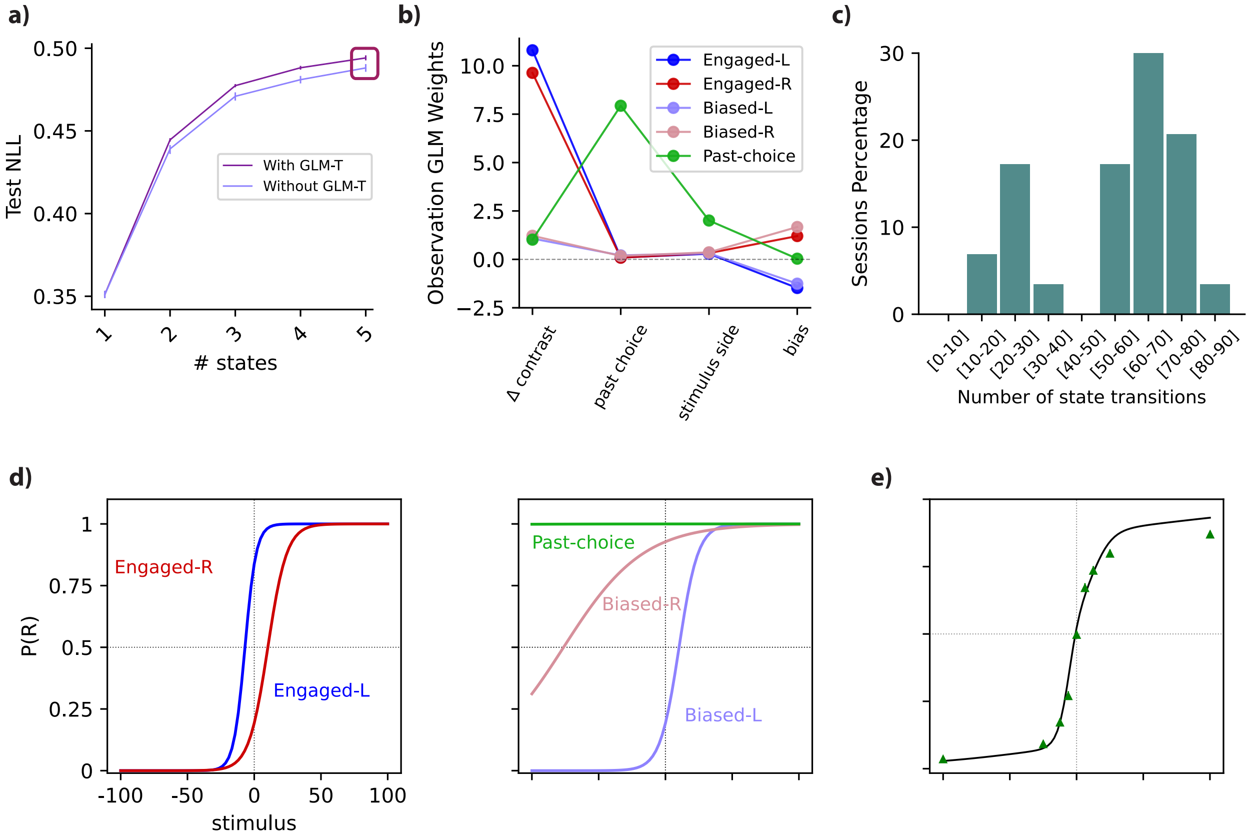

Fig. S1: **Analysis of 5-state GLM-HMM. (a)** A difference emerges when examining the normalized log-likelihood (NLL) between two models for 5-state GLM-HMM: one without the GLM-T, and the other integrating both GLM-O and GLM-T. We used pooled data from all 37 animals in our dataset. The NLL demonstrates an enhancement in performance for the 5-state model that includes GLM-T. We employed and extended Bayesian State Space Modeling (SSM) package to implement this modeling. Data are presented as mean test log-likelihood; error bars denote 68% bootstrap confidence intervals across cross-validation folds (5-fold). **(b)** In the 5-state GLM-HMM tailored to the entirety of IBL data, the inferred observation weights within the model unveil distinct situations. Similar to the four-state model, state 1 is denoted as the Engaged-L state, marked by a notable stimulus (Δ contrast) weight and a moderate left bias weight and state 2, identified as the Engaged-R state, exhibits significant stimulus weight and a moderate right bias weight. In contrast, State 3 (Biased-L) and State 4 (Biased-R) are characterized by reduced stimulus weights, with the bias weights inducing pronounced leftward and rightward biases, respectively. State 5, Past-choice, primarily encodes the influence of the previous choice, as evidenced by a substantial weight assigned to this regressor. Additionally, state 5 assigns comparatively lower weights to the stimulus and the previous stimulus in its representation. **(c)** In this histogram, we observe how sessions surpassing a duration of $T = 821$, the average length of the animal's sessions, showcase the distribution of inferred state changes per session for the 5-state model. **(d)** Analyzing psychometric curves within the context of the GLM-HMM with 5-state. Notably, the psychometric curves for the Biased-L and Biased-R states show gentler slopes, indicating distinct leftward and rightward biases, respectively. Also, there is a flat line for the Past-choice state. **(e)** The standard psychometric curve, the solid black line, represents this mouse's choices. It is formed by combining five per-state curves based on its choice data and by creating a dataset that unfolds over time to match the trial count of the reference mouse. It emerges as a composite representation, resulting from the amalgamation of these individual state curves. Using green triangles, we visually represent the mouse's experimental choice data. Source data are provided as a Source Data file.

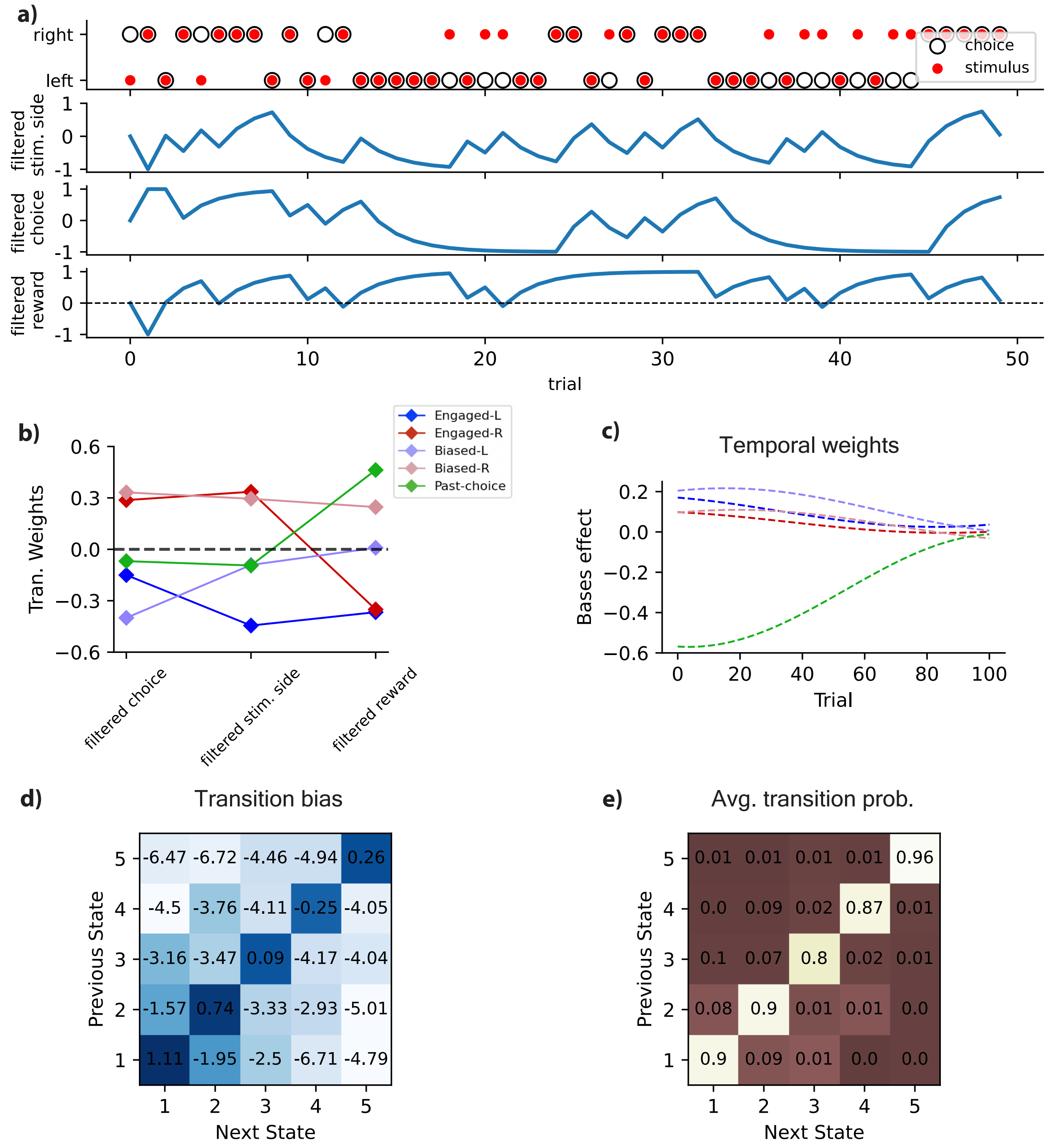

Fig. S2: **Transitional and temporal dynamics of 5-state GLM-HMM. (a)** Plotting the animal choice, stimulus, and filtered covariates for the design matrix (filled red circles and open circles denote the stimulus and the choice, respectively). **(b)** Transition weights associated with the multinomial GLM for the 5-state model, fit using pooled data from all 37 animals. Negative weights for the previous choice and previous stimulus side in states 1 and 3 point out to left-biased transitions, while positive weights in right-biased states (2 and 4) suggest a preference for right-oriented choices. Disengaged states (3 and 4) exhibit a positive previous reward weight, contrasting with engaged states (1 and 2), where the stimulus holds sway, resulting in a negative previous reward weight. Also, state 5 demonstrates a substantial weight assigned to the previous reward, while assigning minimal weight to the previous choice and previous stimulus. This underscores the pivotal role of the previous reward in transitions within the Past-choice state. **(c)** Presenting the temporal patterns of the bases effect. Strategically incorporated, the bases adeptly encapsulate the gradual adaptation of the animal observed during the initial 100 trials of each session, effectively capturing the warm-up effect within the model. **(d)** The transition bias matrix for the GLM-HMM with 5 states. Color encodes transition bias values, zero indicates no effect. **(e)** The deduced transition matrix for the 5-state GLM-HMM shows prominent values along the diagonal, indicative of a pronounced likelihood of persisting within the same state. Color encodes average transition probability values, zero shows no effect. Source data are provided as a Source Data file. Stim., stimulus; Tran., transition; Avg., average.

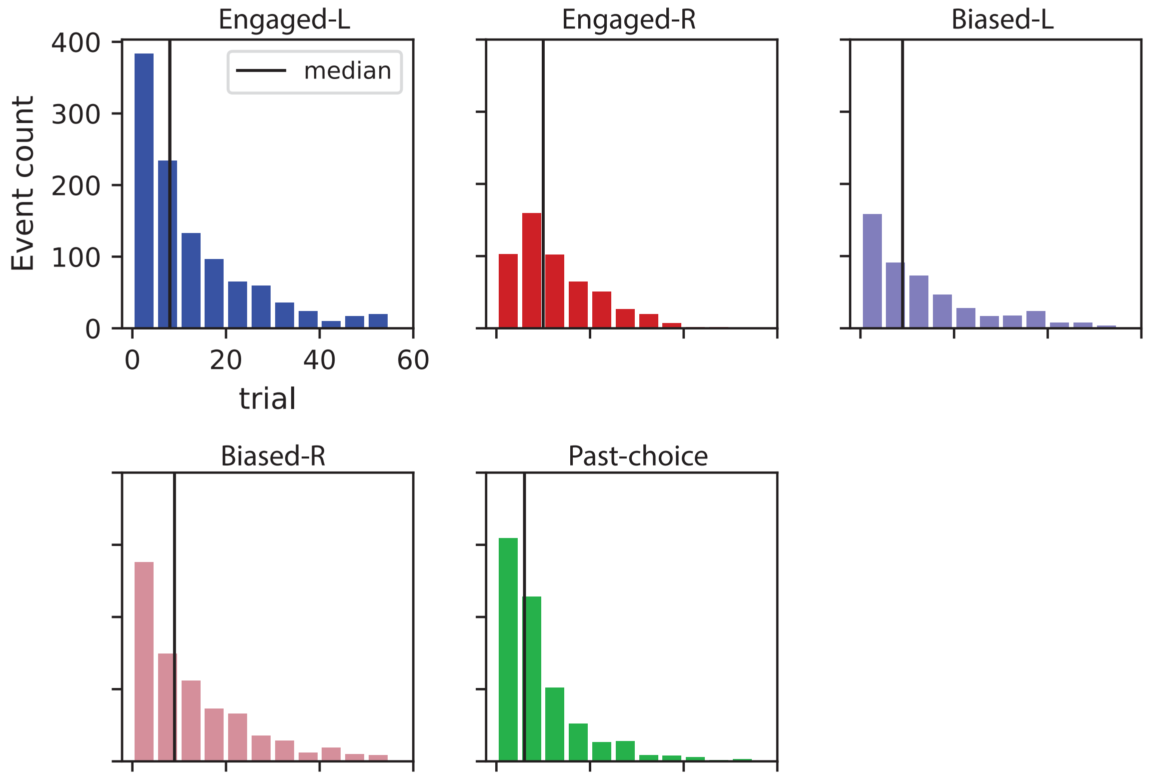

Fig. S3: **Dwell time histogram for the 5-state model.** Utilizing individualized transition matrices, we assessed the projected duration of stays in distinct states for all 37 mice (Axes: x, dwell time (trials); y, count of occurrences). This involved constructing dwell time histograms for the Engaged-L, Engaged-R, Biased-L, Biased-R and Past-choice states, providing a comprehensive exploration of the temporal dynamics within each state for the entire mouse cohort. Source data are provided as a Source Data file.

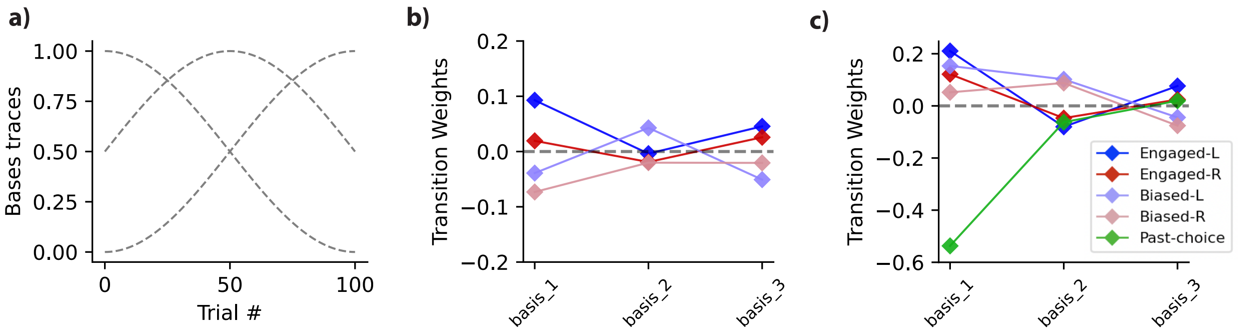

Fig. S4: **Impact of bases transition weights and their effects. (a)** Three bases were introduced into the GLM-HMM to accommodate the animal's warm-up effect observed during the initial 100 trials of each session. **(b)** Transition weights representing the bases effects in the 4-state model (pooled data from 37 IBL animals). **(c)** The similar bases weights for the 5-state model, with basis 1 being more influential in the Past-choice state. Source data are provided as a Source Data file.

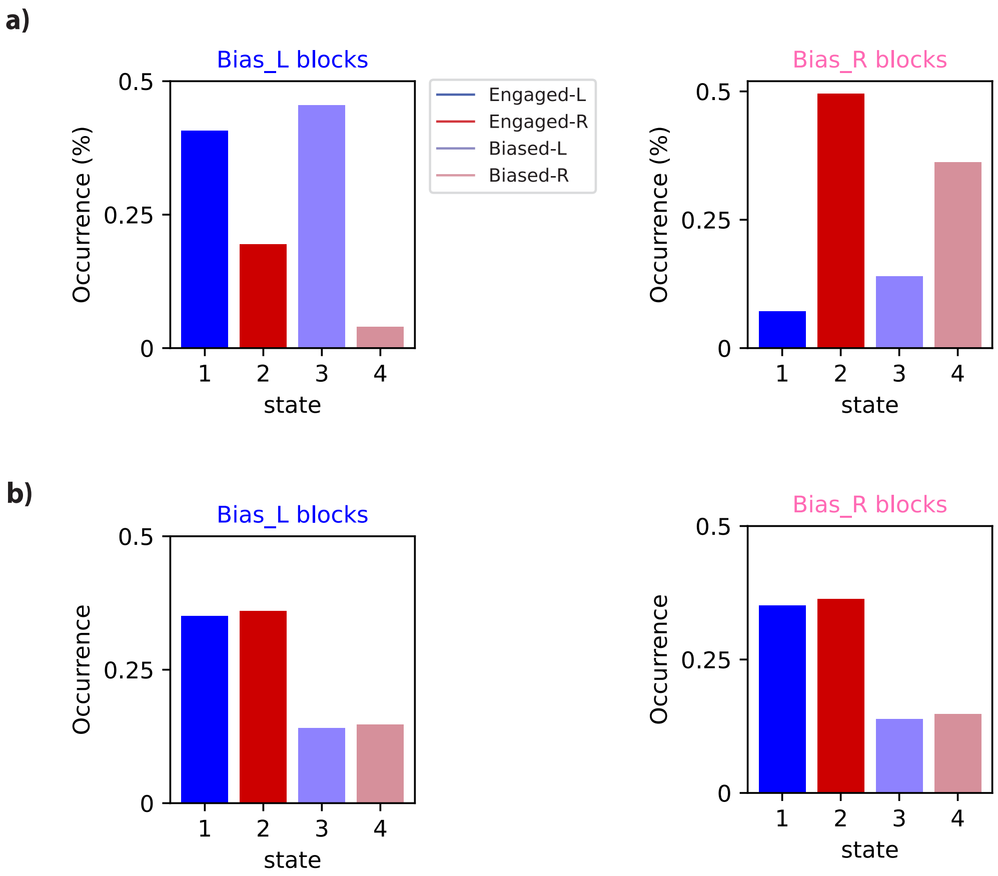

Fig. S5: **Occurrence percentage of models with and without GLM-T for synthetic data.** **(a)** Fractional occupancy of four discrete states using a model with both GLM-T for transition and GLM-O for observation with synthetic data (pooled data from 37 mice). For clarity and precision, we have segregated and presented the results separately for the two distinct types of data blocks: left-biased (Bias-L) and right-biased (Bias-R). This separation allows for a more focused and detailed exploration of how fractional occupancy varies in response to different task conditions. Colors correspond to states as in the main figures (dark blue = Engaged–L; red = Engaged–R; light blue = Biased–L; pink = Biased–R)). **(b)** Fractional occupancy across four distinct states for the GLM-HMM without GLM-T for transition. The outcomes pertain to all trials of the synthetic data, encompassing both right-biased and left-biased data blocks, and are presented separately for each data block type. Source data are provided as a Source Data file.

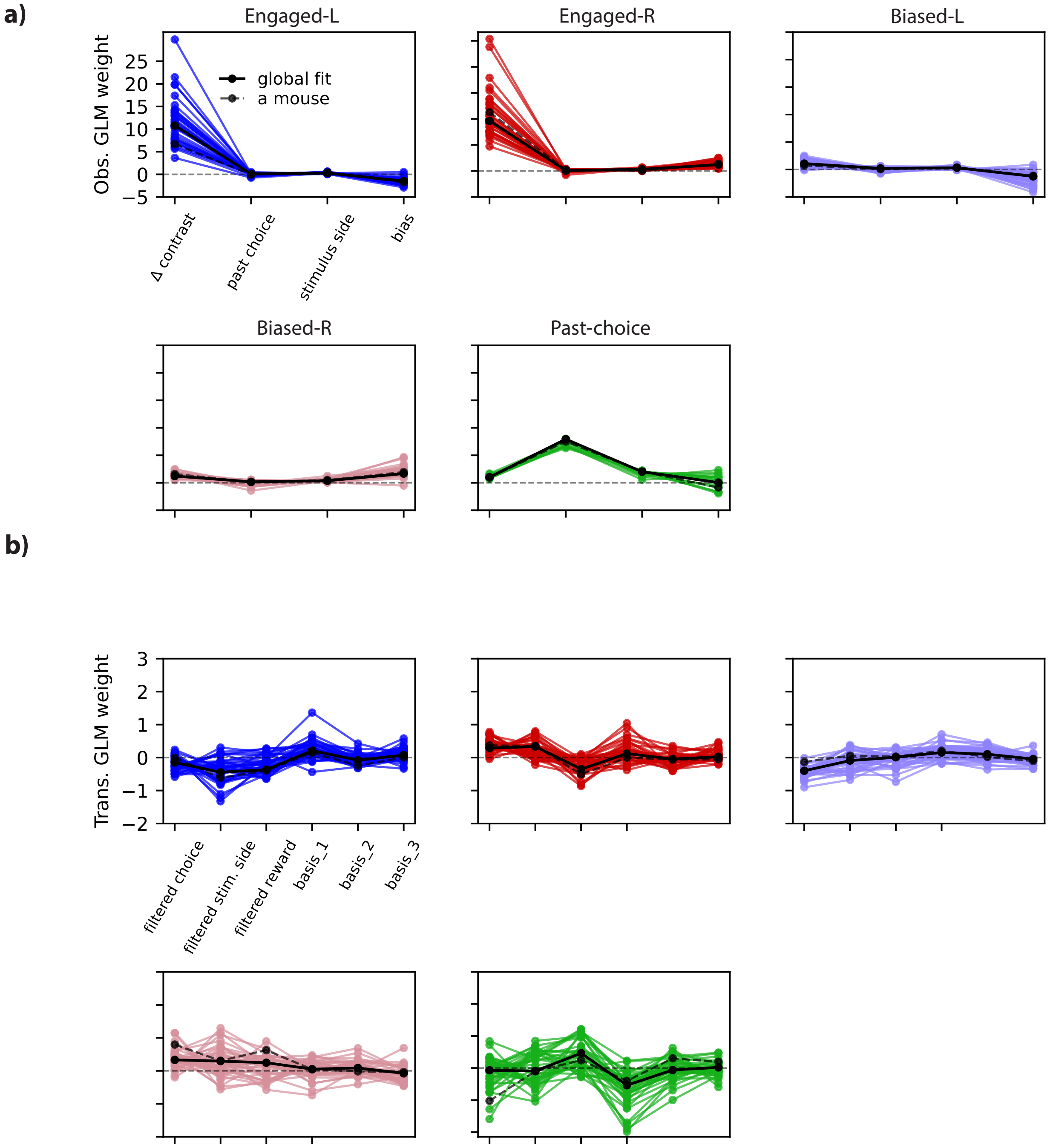

Fig. S6: **GLMs weights of a 5-state model applied to all IBL animals.** Applying the GLM-HMM to choice data from all animals in the IBL dataset resulted in 37 individual fits, one for each mouse where stimulus contrast is shown by Δ contrast. **(a)** and **(b)** show two sets of GLM weights for observation and transition covariates, respectively. The analysis of the 5-state GLM-HMM fits showed that most mice had similar patterns, with distinct states clearly identified. This consistency highlights the reliability of the GLM-HMM framework in capturing these behaviors across the whole group, including both observation and transition weights. **(a-b)**: Unit of study: mouse. $n = 37$ mice (biological replicates; independent animals from the IBL dataset). Each point/curve corresponds to one mouse. Source data are provided as a Source Data file. Obs., observation; Stim., stimulus.

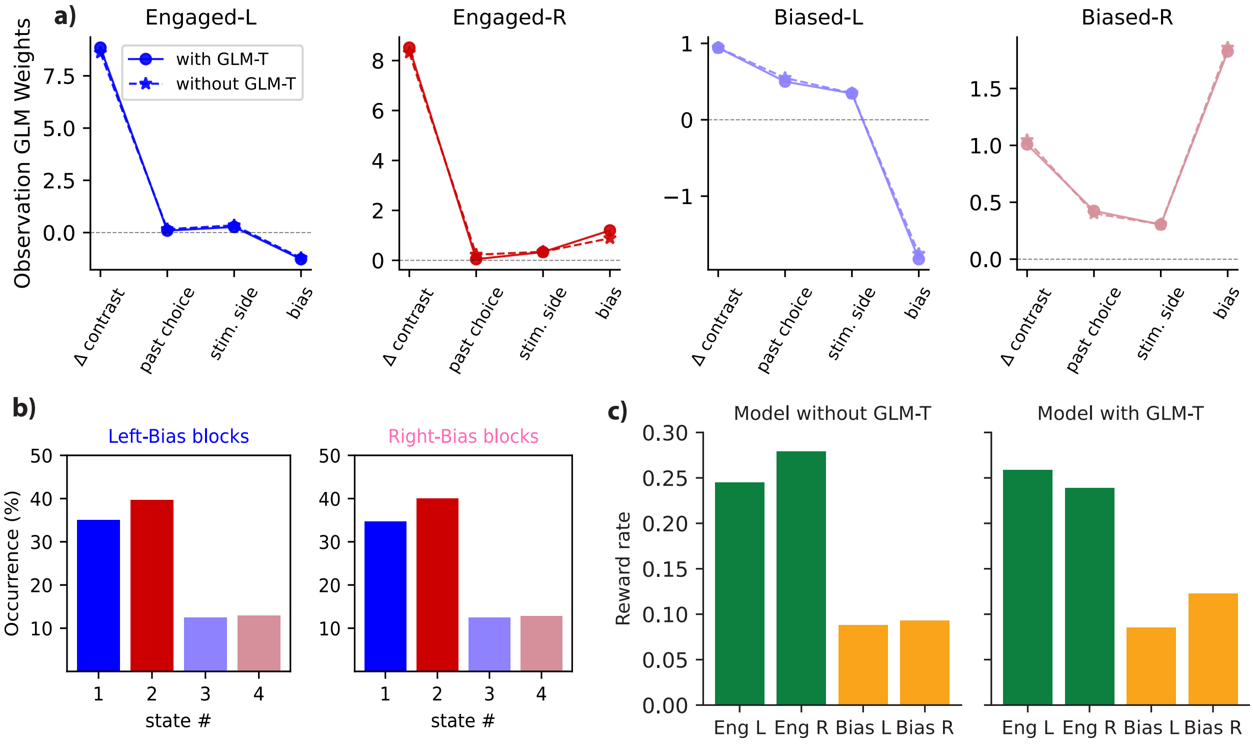

Fig. S7: **Additional analysis and comparison of models. (a)** Observation GLM weights are very similar in models with and without GLM-T, as the addition of GLM-T does not have a significant direct effect on the observation model (pooled data from all 37 mice). **(b)** State occupancy for the model without GLM-T shows that the states do not differ between block types and are not affected by stimulus side probability pattern. **(c)** Comparison of reward rates between models with and without GLM-T shows minimal change, indicating that adding GLM-T does not significantly impact animal choice behavior or reward patterns. Source data are provided as a Source Data file. Stim., stimulus; Eng., Engaged; R., Right; L., Left.

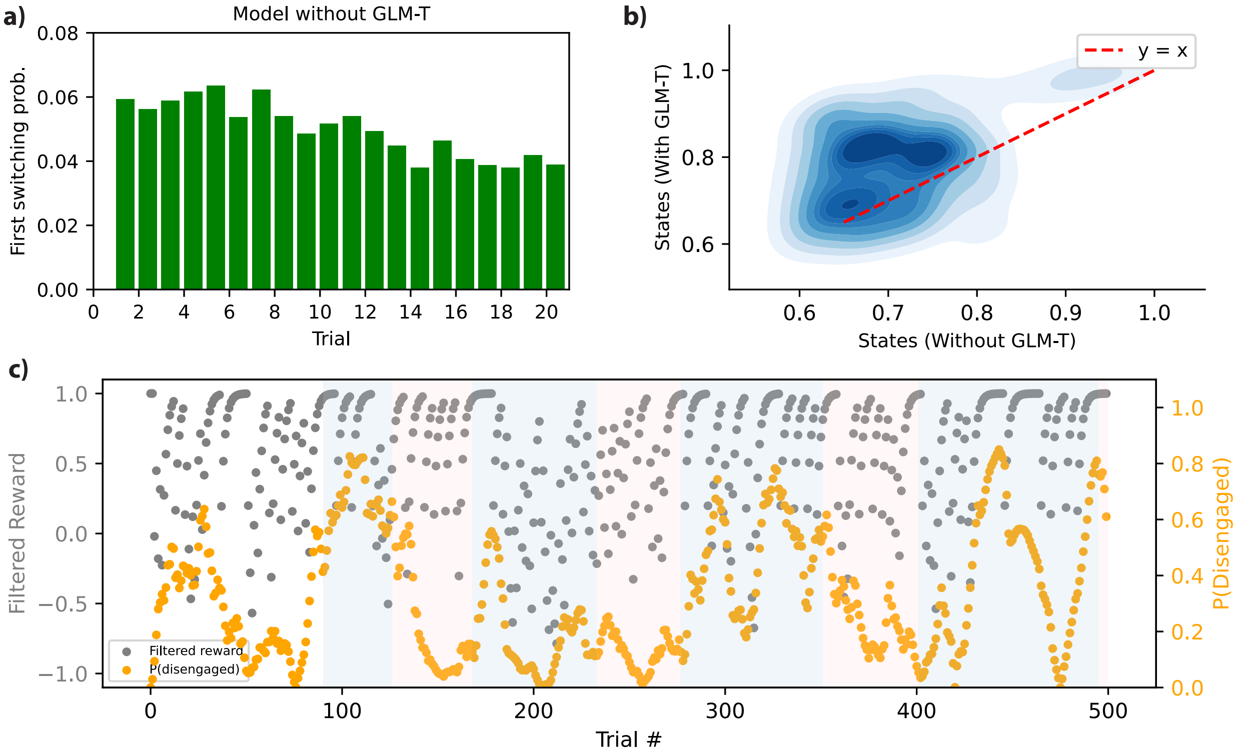

Fig. S8: **Effect of GLM-T on state transitions and behavioral state dynamics. (a)** First switching probability after encountering a biased block in the model without GLM-T, showing a gradual decrease over time. In this model, switching probability does not follow a specific pattern and lacks a clear peak, suggesting that it fails to capture the structured timing of state transitions after encountering a biased block. This indicates that the model may not effectively track behavioral adaptations to block transitions. **(b)** Density plots of inferred states with and without GLM-T (for three representative sessions of Fig. 4a), with most points lying above the $y = x$ line (red dashed), indicating that states inferred by the GLM-T model tend to have higher probabilities than those inferred without GLM-T. **(c)** Relationship between filtered reward (gray) and probability of disengagement (orange) across trials for an example session. The probability of disengagement increases as filtered reward increases, suggesting a correlation between disengagement and reward history. Background shading represents different block types. Source data are provided as a Source Data file. Prob., probability.

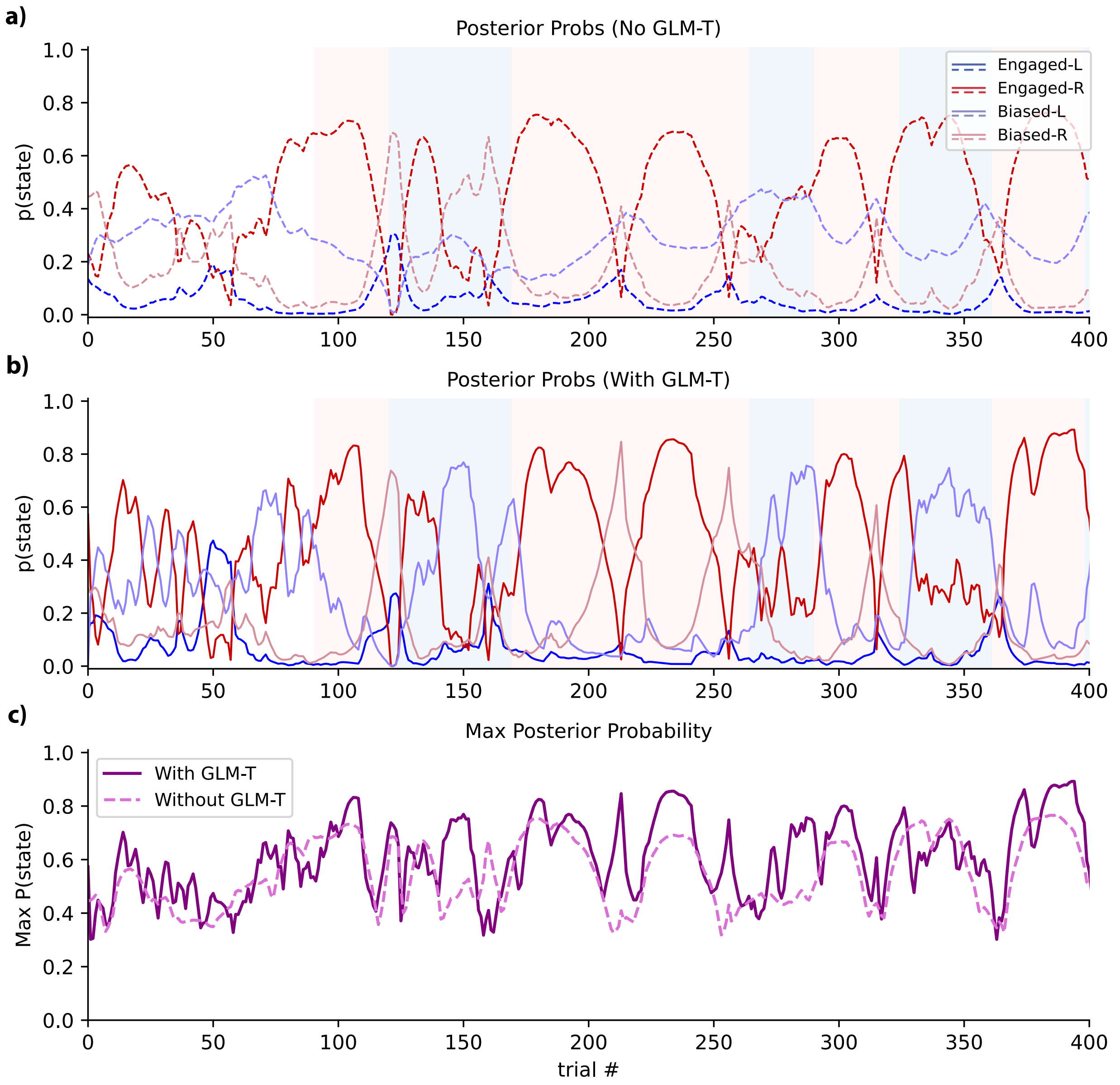

Fig. S9: **Comparison of posterior state probabilities with and without GLM-T for an example session.** **(a)** Posterior state probabilities inferred from the model without GLM-T. **(b)** Posterior state probabilities inferred from the model with GLM-T, displaying smoother and more structured state transitions. Across trials, the GLM-T model shows clearer alignment with block-wise changes in behavior, reflecting more certain and structured state assignments. Background shading denotes task block types, aiding comparison to behavioral shifts. **(c)** Maximum posterior probability from both models for direct comparison. The model with GLM-T (solid line) mostly assigns higher confidence to latent state inferences than the model without GLM-T (dashed line). Confidence is quantified as the maximum posterior probability per trial. Source data are provided as a Source Data file. Probs., probabilities; Max., Maximum.

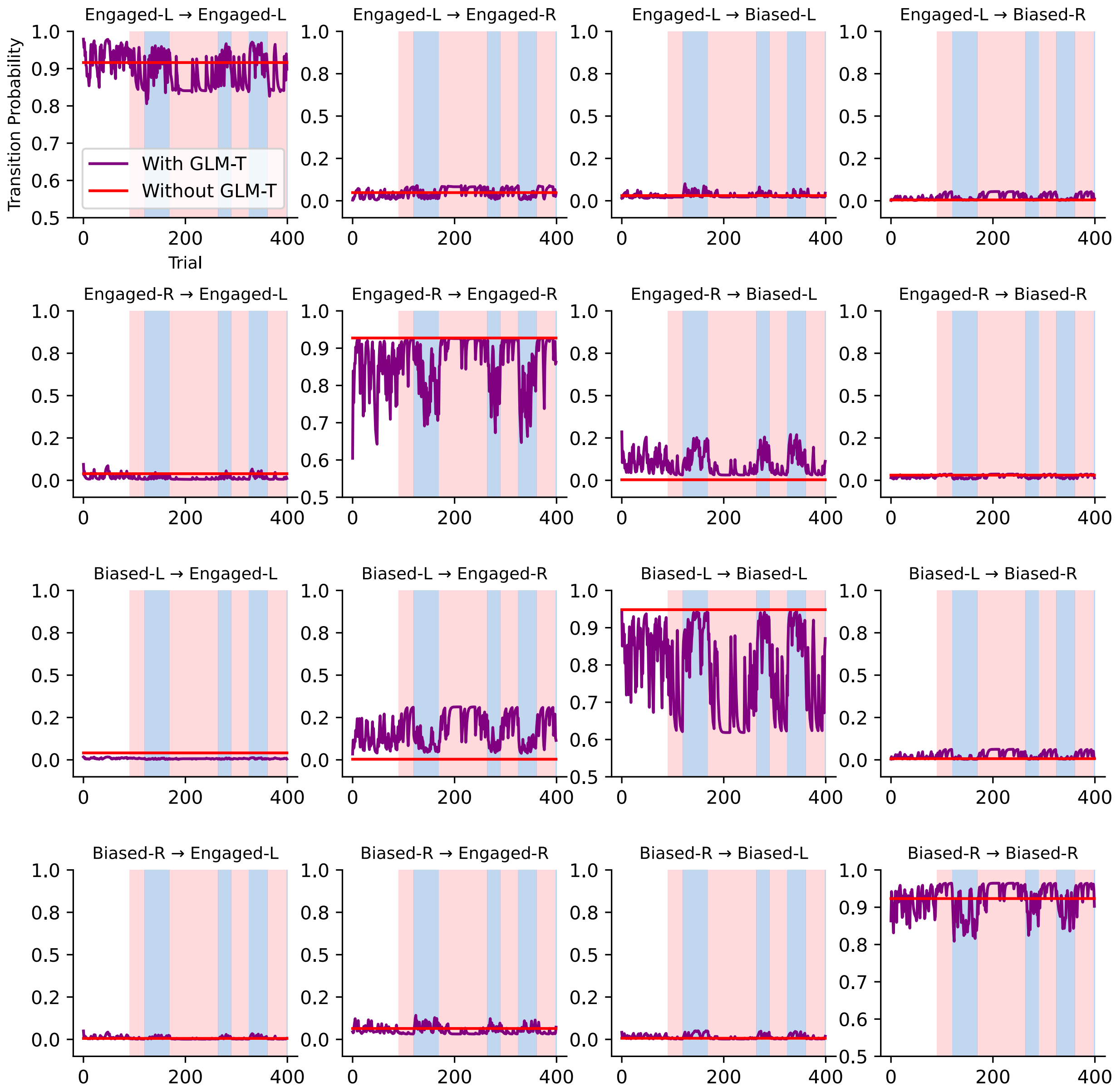

Fig. S10: **Comparison of transition probabilities with and without GLM-T.** Transition probabilities between different states are shown for models with GLM-T (purple) and without GLM-T (red). Each subplot represents a specific transition from one state to another for an example session. The GLM-T model captures dynamic, trial-dependent transitions, showing structured fluctuations aligned with block types (background shading), whereas the non-GLM-T model exhibits flat transition probabilities, failing to capture block-dependent dynamics. Source data are provided as a Source Data file. R., Right; L., Left.

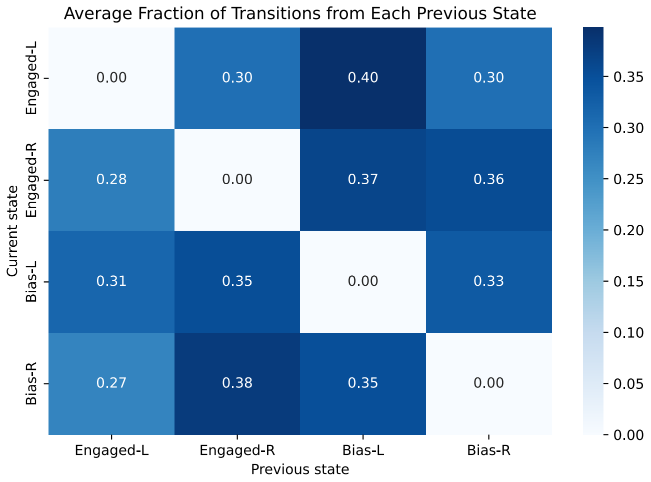

Fig. S11: **Transition probabilities into each state, conditioned on different preceding states in the IBL data.** This heatmap shows that the probability of transitioning into a given state is nearly identical across all possible preceding states, indicating that the model's transition dynamics are not strongly dependent on the previous state. The matrix is averaged across all 37 IBL mice. This confirms that our key conclusions are robust to this modeling choice. Source data are provided as a Source Data file. R., Right; L., Left.
