# Supplementary material for: Identifying the factors governing internal state switches during nonstationary sensory decision-making
Source: Nat Commun. 2025 Nov 27;16:11684. doi: 10.1038/s41467-025-66738-0 (PMC12749821; doi:10.1038/s41467-025-66738-0)
Supplement: Supplementary file 2 — Reporting Summary [file 41467_2025_66738_MOESM2_ESM.pdf]

nature portfolio

Corresponding author(s): Zeinab Mohammadi

Last updated by author(s): Sep 28, 2025

Reporting Summary

Nature Portfolio wishes to improve the reproducibility of the work that we publish. This form provides structure for consistency and transparency in reporting. For further information on Nature Portfolio policies, see our Editorial Policies and the Editorial Policy Checklist.

Statistics

For all statistical analyses, confirm that the following items are present in the figure legend, table legend, main text, or Methods section.

n/a	Confirmed	
☐	☒	The exact sample size (*n*) for each experimental group/condition, given as a discrete number and unit of measurement
☐	☒	A statement on whether measurements were taken from distinct samples or whether the same sample was measured repeatedly
☐	☒	The statistical test(s) used AND whether they are one- or two-sided<br>*Only common tests should be described solely by name; describe more complex techniques in the Methods section.*
☐	☒	A description of all covariates tested
☐	☒	A description of any assumptions or corrections, such as tests of normality and adjustment for multiple comparisons
☐	☒	A full description of the statistical parameters including central tendency (e.g. means) or other basic estimates (e.g. regression coefficient) AND variation (e.g. standard deviation) or associated estimates of uncertainty (e.g. confidence intervals)
☐	☒	For null hypothesis testing, the test statistic (e.g. *F*, *t*, *r*) with confidence intervals, effect sizes, degrees of freedom and *P* value noted<br>*Give P values as exact values whenever suitable.*
☐	☒	For Bayesian analysis, information on the choice of priors and Markov chain Monte Carlo settings
☒	☐	For hierarchical and complex designs, identification of the appropriate level for tests and full reporting of outcomes
☒	☐	Estimates of effect sizes (e.g. Cohen's *d*, Pearson's *r*), indicating how they were calculated

Our web collection on statistics for biologists contains articles on many of the points above.

Software and code

Policy information about availability of computer code

Data collection	No software was utilized for data acquisition, as we worked with a publicly accessible dataset that had already been collected and processed.
Data analysis	The GLM-HMM code and figures for this paper are available at https://github.com/Zeinab-Mohammadi/glm-hmm_final.git. Analyses were performed using Python 3.9, NumPy 1.24.4 and SciPy 1.9.1. We used and extended the Bayesian State Space Modeling framework (version 0.0.1) by Linderman et al. (2020) (available at https://github.com/lindermanlab/ssm), adding new functionality to the GLM-HMM. The modified code, used for model inference, can be accessed through the provided link to our analysis.

For manuscripts utilizing custom algorithms or software that are central to the research but not yet described in published literature, software must be made available to editors and reviewers. We strongly encourage code deposition in a community repository (e.g. GitHub). See the Nature Portfolio guidelines for submitting code & software for further information.

Data

Policy information about availability of data

All manuscripts must include a data availability statement. This statement should provide the following information, where applicable:

- Accession codes, unique identifiers, or web links for publicly available datasets
- A description of any restrictions on data availability
- For clinical datasets or third party data, please ensure that the statement adheres to our policy

We used publicly available behavioral data from the International Brain Laboratory (IBL), which includes male and female mice. Sex was not analyzed, as it was not a

primary focus of this study. The IBL dataset is publicly available at https://doi.org/10.6084/m9.figshare.11636748. To facilitate efficient data access, please see https://int-brain-lab.github.io/iblenv/notebooks_external/data_download.html and use the data tag "2023_Q1_Mohammadi_et_al".

Research involving human participants, their data, or biological material

Policy information about studies with human participants or human data. See also policy information about sex, gender (identity/presentation), and sexual orientation and race, ethnicity and racism.

Reporting on sex and gender	*Use the terms sex (biological attribute) and gender (shaped by social and cultural circumstances) carefully in order to avoid confusing both terms. Indicate if findings apply to only one sex or gender; describe whether sex and gender were considered in study design; whether sex and/or gender was determined based on self-reporting or assigned and methods used. Provide in the source data disaggregated sex and gender data, where this information has been collected, and if consent has been obtained for sharing of individual-level data; provide overall numbers in this Reporting Summary. Please state if this information has not been collected. Report sex- and gender-based analyses where performed, justify reasons for lack of sex- and gender-based analysis.*
Reporting on race, ethnicity, or other socially relevant groupings	*Please specify the socially constructed or socially relevant categorization variable(s) used in your manuscript and explain why they were used. Please note that such variables should not be used as proxies for other socially constructed/relevant variables (for example, race or ethnicity should not be used as a proxy for socioeconomic status). Provide clear definitions of the relevant terms used, how they were provided (by the participants/respondents, the researchers, or third parties), and the method(s) used to classify people into the different categories (e.g. self-report, census or administrative data, social media data, etc.) Please provide details about how you controlled for confounding variables in your analyses.*
Population characteristics	*Describe the covariate-relevant population characteristics of the human research participants (e.g. age, genotypic information, past and current diagnosis and treatment categories). If you filled out the behavioural & social sciences study design questions and have nothing to add here, write "See above."*
Recruitment	*Describe how participants were recruited. Outline any potential self-selection bias or other biases that may be present and how these are likely to impact results.*
Ethics oversight	*Identify the organization(s) that approved the study protocol.*

Note that full information on the approval of the study protocol must also be provided in the manuscript.

Field-specific reporting

Please select the one below that is the best fit for your research. If you are not sure, read the appropriate sections before making your selection.

☒ Life sciences ☐ Behavioural & social sciences ☐ Ecological, evolutionary & environmental sciences

For a reference copy of the document with all sections, see nature.com/documents/nr-reporting-summary-flat.pdf

Life sciences study design

All studies must disclose on these points even when the disclosure is negative.

Sample size	We analyzed data from 123 mice, focusing on sessions from 37 of them in the IBL decision-making task, each of which contributed thousands of trials. In fact, we were able to successfully recover the generative parameters of a GLM-HMM with fewer trials than this. This provided greater statistical power compared to similar studies and was sufficient for our analyses.
Data exclusions	Only animals with sufficient trial data and sessions of acceptable quality were considered in the final analyses presented in the paper. Animals with fewer than 30 sessions or sessions with a high number of error trials, where the animal failed to make a choice or timed out, were excluded from the analysis.
Replication	The study analyzed data collected from multiple animals, with experiments repeated across 123 animals and narrowed down to 37 for our analysis in the IBL dataset. So the experiment presented in the paper was based on data from these animals, with the analysis spanning tens of sessions per animal. The effects identified by the model were consistent across subjects, and the analysis code is freely available to support replication.
Randomization	In the randomization process, sessions were assigned to folds for each animal using a randomized approach during cross-validation. This ensured that data was evenly distributed across folds, minimizing bias and enhancing the robustness of the results. Each animal's sessions were independently randomized to maintain the integrity of the process.
Blinding	Blinding was not applied in this study, as sessions from all animals meeting the criteria were included in the analysis. The use of cross-validation ensured the results were unbiased and reliable, regardless of how the data was divided. Given the comprehensive and systematic approach used, blinding was not required.

Reporting for specific materials, systems and methods

We require information from authors about some types of materials, experimental systems and methods used in many studies. Here, indicate whether each material, system or method listed is relevant to your study. If you are not sure if a list item applies to your research, read the appropriate section before selecting a response.

Materials & experimental systems

n/a	Involved in the study
☒	☐ Antibodies
☒	☐ Eukaryotic cell lines
☒	☐ Palaeontology and archaeology
☒	☐ Animals and other organisms
☒	☐ Clinical data
☒	☐ Dual use research of concern
☒	☐ Plants

Methods

n/a	Involved in the study
☒	☐ ChIP-seq
☒	☐ Flow cytometry
☒	☐ MRI-based neuroimaging

Plants

Seed stocks	*Report on the source of all seed stocks or other plant material used. If applicable, state the seed stock centre and catalogue number. If plant specimens were collected from the field, describe the collection location, date and sampling procedures.*
Novel plant genotypes	*Describe the methods by which all novel plant genotypes were produced. This includes those generated by transgenic approaches, gene editing, chemical/radiation-based mutagenesis and hybridization. For transgenic lines, describe the transformation method, the number of independent lines analyzed and the generation upon which experiments were performed. For gene-edited lines, describe the editor used, the endogenous sequence targeted for editing, the targeting guide RNA sequence (if applicable) and how the editor was applied.*
Authentication	*Describe any authentication procedures for each seed stock used or novel genotype generated. Describe any experiments used to assess the effect of a mutation and, where applicable, how potential secondary effects (e.g. second site T-DNA insertions, mosiacism, off-target gene editing) were examined.*
